# The Quarantine “Irregularities”

**Published:** 1902-03

**Authors:** 


### TEXAS MEDICAL JOURNAL.

AUSTIN, TEXAS.

A MONTHLY JOURNAL OF MEDICINE AND SURGERY.

EDITED AND PUBLISHED BY
F. E. DANIEL, M. D.

ASSOCIATE EDITOR:
WITTEN BOOTH RUSS, M. D.,
San Antonio, Texas.

Published Monthly at Austin, Texas. Subscription price $1.00 a year in advance.

Eastern Representative: John Guy Monihan, St. Paul Building, 220 Broadway, New York City.

Official organ of the State Association of Health Officers, the West Texas Medical Association, the Houston District Medical Association, the Austin District Me dical Society, the Brazos Valley Medical Association, the Galveston County Medical Society, and several others.

The Quarantine "Irregularities."—It is now said that the shortage in the quarantine funds will far exceed the amount reported by the expert accountant, and will reach ten thousand to twelve thousand dollars, or even more. Dr. I. J. Jones, the defaulting secretary, is thought to be in Mexico, and was recently reported to be keeping books for a mining company in the State of Durango. It is generally known that he "blew in" this large sum of the State's money gambling; and his wife and several children are in Austin, reported to be in a destitute condition. The Investigating Committee appointed by the last Legislature to overhaul the books of all the departments are at this writing, going over the Quarantine Department books. They will go back of the Jones defalcation, and look into the matter of the purchase of the bark Rhizetto, for a fumigating plant, which created some stir a year or so ago. The Rhizetto is at the bottom of the bay, meantime, it having been sunk during the storm of September, 1900. The Governor is very anxious to locate Dr. Jones. Dr. Blunt appeared before the committee on the 24th of February. He "courts the fullest investigation." It will be interesting to know who, if anybody, is responsible to the State. *Nous verrions.*

A Suit for Damages for $49,075 was recently tried in the Austin district court, the defendants being the well known firm of Drs. T. D. Wooten & Sons, of Austin. One William Welmer broke the left femur very high up. Dr. Goodall Wooten, with the assistance of Drs. Hamilton and Graves, adjusted the fracture and applied proper splints and weights for extension. The measurements were all right up to the eleventh day, when the attending physician found the weight removed and the dressings loosened. On measurement, there was found to be one and one-half inches shortening. About the fiftieth day, the physician was discharged, and another one called to take charge of the case. The result was great deformity and shortening. Welmer was operated on, later, by Thompson, of Galveston, for correction of the deformity, and the ends of the bones, having been freshened, were screwed together with silver screws. They "pulled out," and Welmer died of septicæmia. The family brought suit against the Wootens for $49,075 damages. The jury deliberated about ten minutes, and brought in a verdict for the defendants; upon which happy and just termination of this vexatious suit the Journal congratulates the Drs. Wooten.

